# Ranking insertion, deletion and nonsense mutations based on their effect on genetic information

**DOI:** 10.1186/1471-2105-12-299

**Published:** 2011-07-22

**Authors:** Amin Zia, Alan M Moses

**Affiliations:** 1 Department of Cell & Systems Biology, University of Toronto, 25 Willcocks Street, Toronto, Ontario, M5S 3B2, Canada

## Abstract

**Background:**

Genetic variations contribute to normal phenotypic differences as well as diseases, and new sequencing technologies are greatly increasing the capacity to identify these variations. Given the large number of variations now being discovered, computational methods to prioritize the functional importance of genetic variations are of growing interest. Thus far, the focus of computational tools has been mainly on the prediction of the effects of amino acid changing single nucleotide polymorphisms (SNPs) and little attention has been paid to indels or nonsense SNPs that result in premature stop codons.

**Results:**

We propose computational methods to rank insertion-deletion mutations in the coding as well as non-coding regions and nonsense mutations. We rank these variations by measuring the extent of their effect on biological function, based on the assumption that evolutionary conservation reflects function. Using sequence data from budding yeast and human, we show that variations which that we predict to have larger effects segregate at significantly lower allele frequencies, and occur less frequently than expected by chance, indicating stronger purifying selection. Furthermore, we find that insertions, deletions and premature stop codons associated with disease in the human have significantly larger predicted effects than those not associated with disease. Interestingly, the large-effect mutations associated with disease show a similar distribution of predicted effects to that expected for completely random mutations.

**Conclusions:**

This demonstrates that the evolutionary conservation context of the sequences that harbour insertions, deletions and nonsense mutations can be used to predict and rank the effects of the mutations.

## Background

Genetic variations contribute to normal phenotypic variation [1]. For human, it is estimated that there are more than 10 million SNPs (i.e. 1 in 300 base pairs on average) with an observed minor allele frequency of ≥ 1% in the population [2]. Recent advances in sequencing technologies [3] have enabled rapid discovery of other types of variations, including mutations expected to have very large effects on protein function such as frame shifting insertions and deletions (indels) and nonsense mutations (mutations that introduce premature stop codons). Amazingly, insertions and deletions are also abundant in the human genome with sizes ranging from single to several million base pairs (bp) [4,5]. For example, in 179 human genomes there were 1.13 million short indels identified [6] indicating an estimate of 1 million indels per human genome (1 in 3600 bps on average). Similarly, recent studies have revealed a surprising number (on average, 80-100) of nonsense mutations per genome observed [6]. Consistent with these data from humans, recent studies in model organisms such as yeast have also revealed an abundance of variants that would be expected to have large effects on phenotype [7,8].

Large-effect mutations (defined here as frame-shift causing indels, nonsense mutations and indels that disrupt highly conserved non-coding DNA) are often assumed to have significant functional impacts, and therefore are likely to cause disease. However, discovery of a large numbers of these variants suggest that many of them might in fact have no (or little) impact on function [9]. A quick survey of dbSNP [10] reveals that of 8311 nonsense mutations (NM) and 33285 frame-shifting (FS) indels have been identified in humans, of which only 2234 (27%) NMs and 801 (2.4%) FS indels have been clinically studied (i.e. have records in OMIM [11] and LSDB [12] and are associated with diseases). Thus, identification of the fraction of these variations that contribute to diseases from those with little effect on function is a great practical challenge [13,14].

Even in cases where the variations are detected in individuals with diseases, identification of the causative variations is a major challenge. For instance, there are 2729 structural variants (including indels) catalogued in the COSMIC project [15] that are potentially associated with lung cancer. Similar observations can be made at the level of individual important disease genes: studies of TP53 (mutated in various kinds of cancer) have identified 1256 somatic, 127 cell-line and 36 germline FS indels [16] as well as 95 somatic, 49 cell-line and 15 germline NMs. Similarly, there are 301 FS indels as well as 160 NMs identified in CFTR (a single mutant gene that is identified to be associated with Cystic Fibrosis [17]). In all these cases, the identification of causative variations in the list of potential candidates remains a challenge.

As the number of observed mutations increases, it quickly becomes infeasible for researchers to manually assess the impact of each one in laboratory. It is therefore becoming absolutely essential to rank the effect of these variations in terms of their impact to define priority in clinical research as well as to weight their effects in association analyses.

There is a wide range of computational methods that predict the effect of SNP on protein function (for a survey of these methods see [18] and references therein). Despite existing interest, to the extent of our knowledge, none of these methods are able to deal with indels and NMs. Here we propose to use evolutionary conservation principles to rank the effect of these variations on genetic information.

Evolutionary conservation has been previously used for predicting the effect of SNPs on protein function [19,7] as well as on non-coding DNA [7]. In SIFT [19] the conservation of amino-acid residues are measured using protein sequence homology. In this method, a non-synonymous SNP (nsSNP) that substitutes a highly conserved residue (i.e. a residue that is less observed in an alignment of homologue sequences) is predicted to have a more deleterious effect. Similarly, in the case of non-coding DNA, in the so-called LR test [7], a SNP is predicted to have more deleterious effect if it causes greater change in the rate of evolution of the DNA site that it alters.

We argue that the underlying ideas used in these methods, i.e. using the evolutionary conservation context [20,21] of the sequence that harbours the mutation for assessing the mutation impact, can be extended to rank the net effect of indels and NMs. In particular, for the protein coding sequences, we propose a scoring scheme that measures the amount of the loss of protein "*information content" *[22] caused by a NM or FS indel. We expect the variations that interfere with conserved residues of a protein to a larger extent to have more deleterious effects. For the non-coding DNA, we propose to use a likelihood-ratio scoring scheme to measure the conservation of the DNA bases that harbour the indels. We argue that indels that fall in highly conserved DNA sites are expected to have more deleterious effects.

To evaluate our hypothesis, we study the effect of indels and NMs in a population of *Saccharomyces cerevisiae *(*S.cer*) yeast [8]. We provide evidence that mutations that disrupt the most highly conserved regions segregate at significantly lower allele frequencies. The paucity of variations in lower allele frequencies suggests that highly deleterious mutations have been removed from the population [23].

Next, we assess the effects of FS indels as well as NMs on human proteins [10,6,12]. We show that variations with no disease association tend to cause less information loss than those associated with disease, suggesting that variations not associated with disease are likely to have less deleterious effects. We further show that NMs that cause higher information loss in human proteins segregate significantly in lower allele frequencies suggesting that not all NMs have the same deleterious effects. We argue that the scoring scheme that quantifies the information loss can be used to rank the effect of mutations in human population.

## Results

### Large-Effect mutations in protein coding DNA

SIFT [19] predicts a non-synonymous SNP to be deleterious if it disrupts a highly conserved amino acid residue in a protein sequence, where the conservation of any residue is measured using an alignment of homologous protein sequences.

We propose that the same principle applies to variations that affect more than one amino acid residue. We know that a FS indel changes the translated amino acid residues from its position to the end of the protein, and a NM causes a premature termination of the amino acid sequence. Thus, in order to extend the approach taken in SIFT, we simply add up the effects of each residue affected. In doing so we are assuming that changes in amino acid residues contribute independently to the overall function of the protein, obviously an over-simplification (see Discussion).

Scores used in SIFT are based on a "normalized" transition probability matrix (TPM) that is built using an alignment of protein sequences homologous to the target protein. The TPM is not suitable for adding the effect of mutations on multiple residues because each column is normalized such that for any residue, the most likely transition is normalized to 1. Since the absolute maxima of the observed probabilities are lost in the normalization, the scores cannot be used to compare the residues or, in our case, to add the effect of substitutions for a number of residues together.

Therefore, we modify the scoring method used in SIFT by using the conventional definition of information carried by biological sequences (e.g. [22]) (for a review see [24]). According to this definition, protein residues that are important for the species are evolutionarily conserved and therefore have a statistically different distribution compared to the freely evolving residues that are under no selection. Biological information corresponds to the difference between the distribution of conserved and non-conserved (freely evolving) sequence. We quantify this difference as follows.

Let us consider a multiple alignment of n protein sequences with length w (that is w columns). We define the position weight matrix (PWM), f, as follows:

where, for instance, *f*_*1A *_represents the relative frequency of amino acid residue "A" in the 1^st ^column of the alignment covering all the 20 protein amino acids. For a freely evolving set of sequences, this matrix is close to the so-called *background distribution g *of the genome (in the simplest form we assume that all amino acids have the same frequency, i.e. *f*_*iA*_=*f*_*iR*_=...=*f*_*iV*_ = 1/20). However, when the sequence alignment is conserved, the PWM, f, is different from the background distribution. For any residue X_i _in a given protein sequence, X, we measure the information content by the ratio of the likelihood that the residue is generated according to the distribution f_i _and the likelihood that it is drawn from a background distribution g [24]:

where, for instance, X_iA _ = 1 if the residue at i^th ^column is amino-acid residue "A". The score S(X_i_) shows how likely is that the residue X_i _is generated from the distribution f_i _(compared to the background distribution g_i_). This score is closely related to the *relative information *and hence our interpretation of information carried by X_i _[24]. The total score of a sequence, S(X), is defined as the sum of information carried by individual residues.

Now suppose that the residue X_i _is substituted by a residue Y_i_. The amount of change in the information carried at site *i *is given by:

When D_i _> 0 we say that the residue i has lost information compared to the original sequence. In rare cases that we observe an increase in the score, i.e. D_i _< 0, we conclude that the genetic event has increased the information content of the residue, i.e. it has a beneficial effect for the gene. The total loss of information, D, of the sequence is defined as a normalized sum of information losses due to individual residues:

By combining the effects of change in individual residues we assume that residues contribute independently to the information loss (or gain) of the sequence. We normalize the loss of information to the total score, S (of the wild-type (WT) sequence), to obtain a dimensionless quantity that can be used to compare the information loss between protein sequences.

Our predictions are based on the information loss score D which defines a normalized ratio of the information content S of the protein without (i.e. the WT) and with the mutation (mutant). The larger the score D is the greater is the information loss due to the mutation. We use this score to rank the effect of mutations.

**Figure 1:**
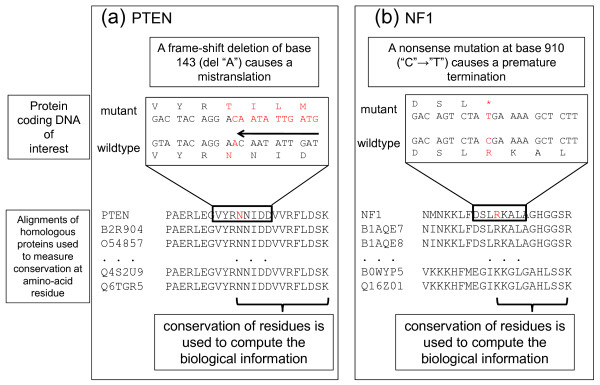
**Computation of the information loss score for variations in the protein-coding DNA**. (a) Deletion of DNA base "A" at site 143 of the human protein PTEN causes a reading frame-shift (depicted by an arrow to the left on DNA sequence) that results in the mistranslation of all residues after the mutation. An alignment of homologous proteins is used to measure the biological information content of the reference sequence (PTEN in the figure) and to measure the loss of information due to this mistranslation. (b) A NM at the DNA site 910 on the human protein NF1 causes a premature stop codon at the residue 304 (denoted by *). The mutant protein is missing residues after the premature stop codon.

As an example, consider the human proteins NF1 [10]. A NM at DNA base 910 ("C" → "T") of NF1 is associated with neurofibromatosis type-I [25] (see Figure 1). The premature stop codon caused by this mutation cuts short the protein at residue 304. The information loss due to this mutation is proportional to the sum of information that was carried by residues that are not a part of the translated amino acid sequence any more. Similarly, a FS deletion at site 143 of the human protein PTEN [10] has been shown to be associated with a type of skin cancer [26]. The amino acid residues after the shift caused by the deletion are different from their original WTs (Figure 1). The information loss, in this case, is a normalized difference between the S scores of the mutant and the WT sequences. For the case of the NM of G to T at the base 1047 of the TP53 tumour repressor protein [10,16], the score of the mutation is considerably lower, consistent with the location of the mutation at the C-terminus of the protein. Table 1 shows corresponding scores for these three genes.

**Table 1:** examples of mutations in human genes

Gene	cDNA length	Mutation at base	D
NF1	8517	2730 (NM)	0.80

PTEN	1209	429 (deletion)	0.66

TP53	1182	1047 (NM)	0.09

Information loss due to mutations on human genes

### Effect of mutation in non-coding DNA

It is widely accepted that the non-coding DNA harbours many functional elements [27,28] and that variations in these regions can have phenotypic effects and cause disease [29-34]. We use conservation of bases in the non-coding DNA to assess the impact of these variations. While in principle, a similar approach used for computing the information loss in coding regions could also be applied to the non-coding DNA, building sequence alignments of homologous non-coding DNA from distantly related species is infeasible due to the relatively fast rates of evolution.

**Figure 2:**
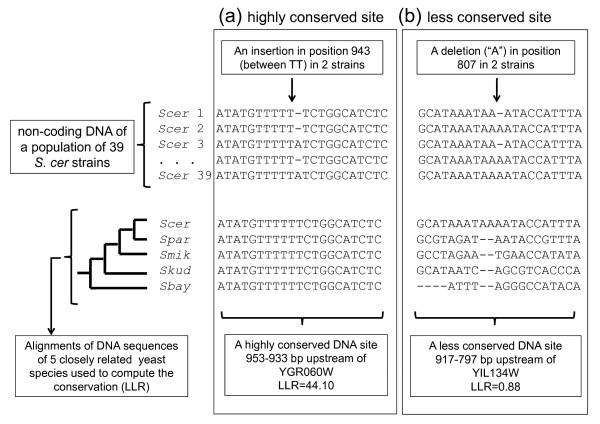
**Computation of the LLR score for indels in the non-coding DNA**. Once the indel is detected in population (here 39 *S. cerevisiae *yeast strains depicted in the top panel), an alignment of width W = 21 of orthologous sequences (referred to as the DNA site) from 5 closely related yeast species (including the reference *S. cer*, *S. paradoxus*, *S. mikatae*, *S. kudriavzevii*, and *S. bayanus*) is made centred around the position of the indel (bottom panel). The conservation of the DNA site is measured through the LLR score by measuring the relative rate of evolution of the site compared to the synonymous rate of evolution of the protein's coding region. (a) shows an insertion that falls into a highly conserved DNA site whereas (b) shows a deletion that falls into a region that is not conserved.

We must therefore use an approach based on sequence alignments from closely related species, where conservation of functional elements in non-coding DNA is detectable [35-38]. We hypothesize that mutations that fall into most conserved sites have more deleterious effects compared to others that do not disrupt conserved regions [39-42] (see Figure 2 for examples of highly conserved and non-conserved non-coding DNA).

Consider 1000 base-pair (bp) wide regions of non-coding DNA upstream of genes. Given an alignment of DNA sequences of width W, we measure conservation of a DNA site by the log-likelihood ratio (LLR) [7,43]:

where*λ*_*syn*_is the average rate of evolution of synonymous mutations in the coding DNA of the protein and  is the maximum likelihood estimate of the rate of evolution of the non-coding DNA site. Here T is the evolutionary tree of the species being used in building the DNA alignments. The LLR measures how fast a DNA site is evolving compared to the synonymous rate of evolution of the protein coding DNA. Slowly evolving sites (i.e. more conserved) give larger LLR values. For these sites, the likelihood (in the LLR numerator) that the site is evolving according to the rate λ^*^ (which is no more than *λ*_*syn*_) is significantly greater than the likelihood that the site is evolving as fast as the synonymous rate of protein coding region. Alternatively, the DNA sites that evolve at a rate similar to the synonymous sites in the coding region are not conserved and therefore give a lower LLR scores.

The results of our analysis provide evidence that LLR score can also be used to measure the effect of indels that disrupt the conserved DNA sites. A similar approach was previously used in [7] to assess the effect of SNPs in non-coding as well as the protein-coding regions [43].

### Genome-wide population analysis with mutations in yeast protein coding regions

We sought to test whether our scores for large-effect mutations reflected their functional impact. More deleterious variants are expected to segregate at lower frequencies in the population and occur at lower densities that would be expected of neutral variants [23]. Therefore, in a natural population, we expect mutations with larger predicted effects to segregate at lower frequencies and be found at lower densities than mutations with smaller predicted effects.

**Figure 3:**
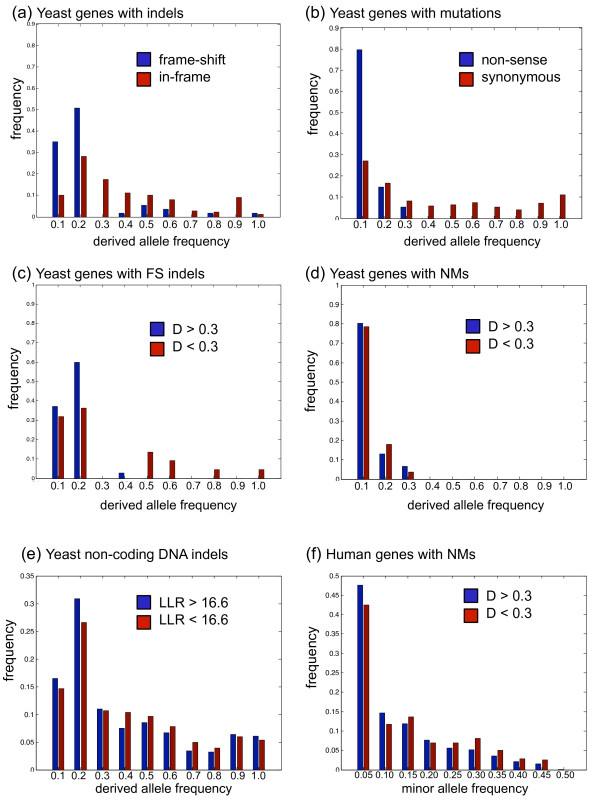
**Spectrums of mutation allele frequencies**. (a) Distribution of yeast frame-shifting indels (blue) skewed to lower DAFs compared to genes with in-frame indels (red) suggesting a relatively greater selective pressure on these genes. (b) Distribution of yeast NMs (blue) skewed towards the lower DAFs compared to the genes with synonymous SNPs (red) suggesting a relatively greater selective pressure. (c) Distributions of yeast FS indels that cause (blue) larger information loss (D > 0.3) and the those that cause (red) lower loss (D < 0.3). (d) In the case of genes with NMs, no mutations with higher allele frequency were observed and therefore the results are inconclusive. (e) Distribution of yeast non-coding DNA sites harbouring indels versus the indels' derived allele frequency (DAF) for two classes of indels: (blue) indels that fall in highly conserved sites (LLR > 16.6) and (red) indels that fall in less conserved sites (LLR < 16.6). The threshold THR = 16.6 is obtained using the Poisson random fields method (see Methods). (f) Distribution of human NMs versus their minor allele frequency in the human population for two classes of mutations causing (blue) greater information loss (D > 0.3) and (red) less information loss (D < 0.3), respectively.

Using sequence data from a population of 39 strains of *S.cer *[8], we identified genes that contain single NM as well as genes with a single FS indel. We first computed the derived allele frequency spectrum (DAF) and tested for a shift towards lower DAFs (left of the spectrum) [23], which is expected under purifying selection [8]. We confirmed that FS causing indels segregate at significantly lower frequencies (Kolmogorov-Smirnov (KS) test, p < 10^-6^) than do in-frame indels (those that do not cause frame shifts) (Figure 3a). Similarly, we found a significant skew to the left for NMs (KS test, p < 10^-6^) suggesting that they segregate at much lower frequency in the population than do synonymous polymorphisms (Figure 3b).

In order to test whether our information-based score differentiates FS indels that are likely to be deleterious from those that are likely to be neutral, we computed the fraction of high frequency alleles defined as DAF > 0.2. We compared this fraction between genes with higher score, i.e. D > 0.3, which we considered to have a greater deleterious effects, and genes with score less than 0.3 (which we considered moderate deleterious effects).

We found, for the case of FS indels (see Figure 3c) that the fraction of high frequency alleles for the genes with D > 0.3 was significantly less than the fraction of high frequency alleles for genes with for the genes with D < 0.3 (1/35 versus 7/22 with Fisher's exact test p = 0.004) (due to sparse spectrums, we did not use the KS test for this case). For the NMs, the results were inconclusive because we did not observe mutations with high allele frequency (Figure 3d). The paucity of high frequency alleles is consistent with stronger purifying selection on the mutations we predicted to be more deleterious (using the score D).

**Figure 4:**
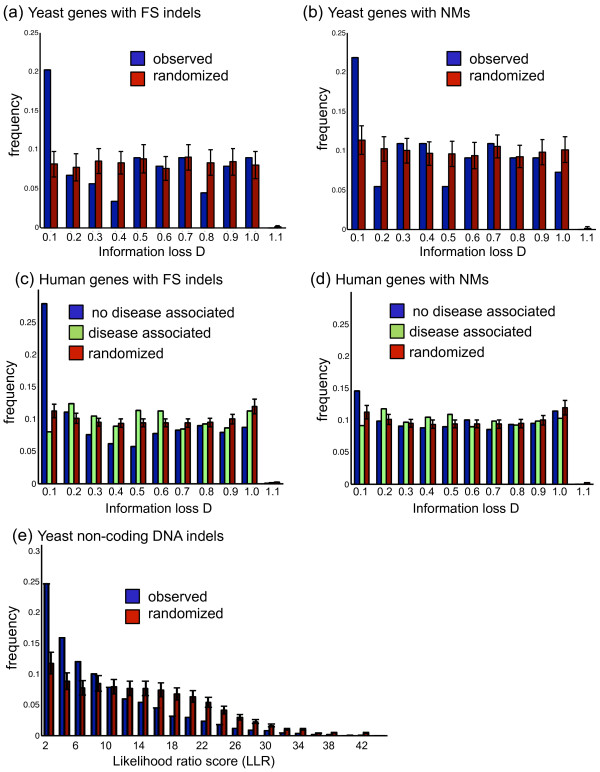
**Randomization experiments**. (a) Distribution of the information loss caused by the FS indels in the yeast population (blue) compared to the density of a set of randomly distributed FS indels throughout the same set of yeast genes (red). (b) Distribution of the information loss caused by the NMs in the yeast population (blue) compared to a set of randomly distributed NMs throughout the same set of yeast genes (red). (c-d) Distributions of FS indels and NMs in the human population (dbSNP [9]), respectively, with respect to the information loss they cause. For each type of variation, this distribution is different when compared to the variations with records of disease association (green) and variations that do not have such records (blue). A set of randomly generated FS indels (red) shows a similar distribution to those that are associated with diseases. (e) The distribution of DNA sites that harbour indels with respect to their LLR score is compared to the distribution of DNA sites randomly chosen from 1000 bp upstream of all genes in the yeast dataset. In panels a and b, the "randomized" histogram bars represent the mean of 100 random samplings of the data, and the error bars represent the standard deviation observed over the 100 samplings, while in panels c-e the "randomized" histogram bars represent the mean of 50 random samplings of the data, and the error bars represent the standard deviation observed over the 50 samplings.

To test for the effects of selection on the density of variations, we compared the distribution of scores to that expected if the mutations were randomly placed. For this purpose, we generated 100 sets of variations randomly placed on genes in our dataset and computed the information loss scores for them. We then compared the distribution of the scores from the 100 random datasets to the distribution of scores obtained from our yeast dataset. As can be seen from Figure 4a and Figure 4b, these distributions are significantly different (KS test with p < 0.05 for both indels and NMs) such that there is a great enrichment of mutations with very small scores, D << 0.1. This suggests that purifying selection has acted to remove mutations with greater score D from the population.

### Genome-wide population analysis of indels in yeast non-coding DNA

We identified indels that fall within the promoter regions (1000 bp upstream) of genes in a population of 39 strains of *S.cer *budding yeast [8]. For each identified indel, we computed the conservation of surrounding DNA site (of width 20 bps) using an alignment of non-coding DNA sequences of orthologues genes from 5 closely related yeast species (see Methods and Figure 2).

We sought to test whether indels that fall in highly conserved DNA sites segregate at lower DAFs compared to those that do not disrupt conserved DNA sites (i.e. that fall in relatively non-conserved regions). For this purpose, we define a LLR score threshold THR = 16.6 for dividing indels into two sets: those that fall into highly conserved DNA sites (i.e. sites with LLR > 16.6) and those that fall in less conserved DNA site (i.e. sites with LLR < 16.6). The threshold THR = 16.6 is estimated such that the difference between the selection coefficients of the two classes is maximal (see Methods for detail of using Poisson random fields for computing the optimal threshold).

Figure 3e shows the DAFs for the two sets of indels. The allele frequency spectrum of indels falling into highly conserved DNA sites is shifted towards lower DAFs significantly (KS-test p = 0.0033) suggesting that these mutations are under stronger purifying selection.

To test for the effects of selection on the density of indels, we compared the distribution of DNA sites with respect to their LLR scores with what is expected if the indels were placed randomly. For this purpose, we generated 50 sets of indels distributed randomly in the 1000 bp upstream of all genes in the reference *S.cer *and computed the LLR scores for the corresponding DNA sites in which they fall. Figure 4e shows that the distribution for DNA sites in our dataset is significantly different than the random dataset (KS test, p < 10^-6^), such that there is a great enrichment of sites with lower LLR scores. This suggests that indels at highly conserved DNA sites have been removed from the population by purifying selection.

### Ranking of mutations in yeast

Our method identifies candidates for new deleterious variations in a pool of genes with mutations. We ranked the mutations in our yeast dataset in terms of their deleterious effects on the corresponding proteins. In the following, we study the top ranked FS indels and the NMs with lowest scores.

Table 2 shows the top 5 FS indels with the highest D scores in our dataset. We observed that three of the genes on the list, that are also essential to yeast [44], carry highly deleterious FS indels. We further studied possible association of these indels with yeast phenotypes using data from phenotypic experiments [45] as well as phenotype data from [46]. One of the FS indels was found in the SMD1 gene. A reduction in the function of SMD1 is associated with a decrease in the resistance of yeast to the drug Tunicamycin [46]. We therefore considered the reproduction efficiency (RE) of yeast strains in the presence of 1.5 μg/mL Tunicamycin [45]. We observed that the RE of the 2 individuals carrying FS indels was lower compared to the population. Specifically, the 2 individuals were among the 8 strains (out of 25) that had REs < -4 (average RE in strains that had genotype data at this locus is -2.81).

**Table 2:** highest and lowest D-scores

a) FS indels				
**Rank**	**Gene**	**Essential**	**Mutation at base**	**D**

1	SMD1	YES	2	0.987

2	RPL8A	NO	224	0.985

3	TFB3	YES	92	0.963

4	RAD34	NO	154	0.963

5	ERG25	YES	214	0.928

b) NMs				

74	TIF5	YES	1213	0.002

73	CRT10	NO	2869	0.002

72	COX19	NO	292	0.001

71	SWI4	NO	3256	0.001

70	HYM1	YES	1193	0.001

A sample of ranked genes in terms of their information loss score D. (a) The top 5 genes with FS indels with highest scores (b) The bottom 5 genes with NMs with lowest scores.

One of the other top ranked indel predictions was in TFB3. A reduction in the function of the gene TFB3 is associated with an increase in resistance to the same drug [46]. Interestingly, we observed the expected effects in the 2 individuals that carry the FS indel (and therefore are predicted to lack the function for this gene). Specifically, the 2 individuals were among the 8 individuals that had REs > -2 (average RE in strains that had genotype data at this locus is -3.06). Because of the small number of individuals that carry the putatively deleterious FS indel alleles (here 2) we were not able to test the significance of these phenotypic observations. However, these examples show the practical uses of the proposed methods.

We further studied the bottom 5 genes with NMs with lowest scores in our dataset. Table 2 shows that while it is possible to observe NMs in essential genes (ranks 74 and 70) [44], our method predicts that these mutations have no substantial effect on the function. These mutations are located in the C-termini of these genes.

### Variations in the human population

To test whether our methods can be applied to the variations in the human population we examined genes with FS indels and NMs reported in dbSNP [10]. We categorized the variations into two classes: variations that have records of diseases association in OMIM [11] and LSDB [12] and variations with no such records. We expect that the latter class (i.e., variations with unknown disease association) to contain mutations with less harmful effects.

We then sought to study the allele frequency spectrum of these variations. Heterozygousity information was only available for the NMs with no disease association. We used the heterozygousity to compute the minor-allele frequency (MAF) of the NMs that were used in the following MAF spectrum analysis. We studied the segregation of NMs in the human population by comparing the spectrums of MAF of the NMs that cause greater information loss (i.e. 1101 NMs with D > 0.3) and the NMs with lower effects (i.e. 505 NMs with D < 0.3). As for the non-coding indels above, we computed D = 0.3 as the optimal threshold using the Poisson random fields method (see Methods). We found that the spectrum of MAF of NMs with higher information loss (Figuure 3f) is significantly skewed to left (KS test, p = 0.002) indicating that these variations segregate significantly at lower allele frequencies. Thus, these mutations appear to have more deleterious effects in the population.

To study the effects of selection on mutation' density, we compared the observed distribution of scores with that expected if variations were randomly placed. To do so, we computed the score D for a large number of FS indels as well as NMs placed randomly on the human genes in our dataset. Figures 4c and 4d compare the distribution of these scores for FS indels and NMs, respectively, with variations in our dataset divided into two classes: with and without disease associations. The insignificant different between distributions for variations with disease association compared to randomly generated variations (KS test, p = 0.09 for FS indels and p = 0.09 for NMs) suggests that these variations are distributed randomly throughout human genes. On the other hand, there is a significant difference between the distributions of variations that have no disease association compared to randomly placed variations (KS test, p < 10^-6 ^for FS indels and p < 10^-4 ^for NMs). Similarly, there is also a significant difference between the distributions of variations with and without disease association (KS test p < 10^-6 ^for FS indels and KS test p = 0.0006 for NMs). The significant abundance of mutations with lower deleterious effects in the data with no disease association, or in other words, the paucity of variations with higher information loss scores, indicates that purifying selection had acted on highly deleterious variations.

### An example for the application of the method to study human genes

**Figure 5:**
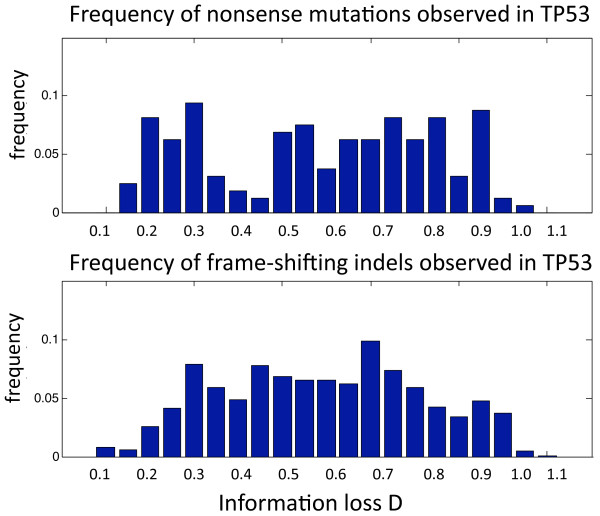
**Distribution of mutations on the human tumour repressor TP53 with respect to their respective loss of information scores**. The upper panel shows the distribution of D for 95 somatic, 49 cell-line and 15 germline NM in the TP53 tumour suppressor. The lower panel show the distribution of D for 1256 somatic, 127 cell-line and 36 germline FS indels reported for TP53 protein. These mutations are associated with a wide range of cancers.

The abundance of variations in genes associated with diseases as well as a wide range of information loss they cause is overwhelming. As an example, consider the tumor repressor gene P53 and its protein product TP53 [47]. There are 95 somatic, 49 cell-line and 15 germline NMs as well as 1256 somatic, 127 cell-line and 36 germline FS indels reported to have association with different types of cancer. These mutations have wide ranges of effects on protein conservation (Figure 5). While it is difficult to determine which mutations cause these diseases [48,49], different effect of these mutations on protein conservation suggests different roles they potentially play in damaging the protein function. For instance, 10 NMs as well as 100 FS indels of these sets with lowest score (D < 0.1) are positioned in residues after 300 in TP53 associated with the exons 9-11. Consistent with the prediction that these have little impact on protein function, these regions are not part of the so-called "hot spots" in this protein (i.e. exons 5-8) [50].

## Discussion

Our proposed methods are useful for practical purposes to sort a huge number of FS indels, NMs, as well as indels in non-coding DNA in terms of their deleterious effect. It is important to note that our methods do not seek to classify variations into deleterious and non-deleterious but rather to rank their effect for further analysis and laboratory experiments.

For the variations in the protein-coding DNA, the proposed score is built upon the principle assumption that the effect of nonsense or FS indel mutations on protein can be computed as sum of effects due to individual residues. This is obviously an over-simplification that is widely accepted in statistically modeling the individual columns of an alignment independently. A more complex method that considered correlations between each residue was also implemented using a profile-HMM based on a generative hidden-Markov model [51] (data not shown here). The score S were computed as the likelihood of the sequence given the profile-HMM [52]. Similar prediction results were observed, i.e. sequences with mutations in more conserved regions resulted in lower scores.

We observed a strong correlation between the position of the nonsense or FS mutations and the loss of information they cause (Additional file 1, Figure S1). We were not able to demonstrate that the D score outperforms the percentage of the protein that is truncated (the "length lost"). When we compare the distribution of the length lost to the random expectation (Additional file 1, Figure S2) we find that the length-lost score appears to show less deviation from the random expectation than the D score for the human data (Additional file 1, Figure S2c,d). This is consistent with the hypothesis that the D score captures more information than the length lost. While simply considering the number of residues affected provides a reasonable guess at the impact of mutations "on average", there are cases for which the position of the mutation does not reflect its effect on evolutionary conservation (off-diagonal points in Additional file 1, Figure S1). Furthermore, we believe that the D score represents a more principled approach to quantifying the importance of these variants because it directly measures evolutionary information, and because it is consistent with previous approaches to quantify the effects of variants, such as SIFT. However, if multiple sequence alignments are not available, the length lost might also provide a reasonable substitute to quantify the effect of a FS indel or NM.

## Conclusions

Identification of causative mutations for diseases remains a challenge even for the case of single genes, let alone in cases where mutations are studied in a network of genes and regulatory elements (e.g. variations affecting genetic pathways). Due to the overwhelming abundance of variations, the information loss score, that captures the evolutionary conservation context of the sequences harbouring mutations, seems to be a good candidate for weighting variations in large-scale association analyses [53,54].

## Methods

### Information loss in protein coding DNA

To obtain the information loss, D, we compute the scores of the WT and the mutant sequences against a position weight matrix as it is defined above. The mutant proteins with highest scores D are more likely to carry a highly deleterious mutation.

The PWM is built using an alignment of protein sequences homologous to the WT protein. We begin with the WT protein sequence as the query to PSI-BLAST [55] with adequate number of iterations (2-5) to collect a set of sequences that are locally homologous to the WT protein (similar to SIFT [19]). The database of protein sequences we used for this study was SWISS-PROT 3.9 [56]. We then remove sequences that are greater than 90% identical to the target sequence from the alignment. This is to maintain a minimum degree of diversity between sequences and to avoid biasing the estimation of the PWM towards closely related species. There are cases where PSI-BLAST did not return sufficient hits to build the alignments. We exclude mutations on genes from our dataset in these cases.

Any column of the PWM, f, is the maximum likelihood estimate of the distribution of amino acid residues observed in the alignment. In ideal cases where there is sufficiently large number of sequences in the alignment, the PWM is simply a matrix with columns equal to the relative frequency of each observed amino acid residue at that column. However, in practice, due to a relatively large number of residues (i.e. 20) compared to the alignment size, there are chances that many residues are not observed and hence their corresponding entry in the PWM is zero. We resolve this issue by considering a minimum number of pseudo-observations for each residue chosen proportionally to the background distribution g. We compute the background distribution, g, by a genome-wide average over all the coding and non-coding regions. The final alignments have always gaps. For each column of the alignment, we uniformly distribute the relative frequency of gaps among all residues.

### Yeast dataset

To validate our prediction method we used complete genome sequences of 39 strains of *S.cer *as our test data. The data, here referred to as SGRP data [8], includes a reference lab strain (S 288c) plus 38 other strains from different sources including other labs, pathogenic, baking, wine, food spoilage, natural fermentation, sake, probiotic, and plant isolates and from a wide range of geographic areas including North America, Europe, Malaysia, West Africa and Asia. The data were sequenced using Sanger sequencing on ABI 3730 DNA sequencers as well as Illumina Genome Analyzer. We used genome-wide alignments made by PALAS (see Supplementary data for [8]). For computation of derived allele frequency of indels and NM, we used a reference *Saccharomyces paraduxos *(*S.par*) as the out-group to the *S.cer *strains.

All DNA base reads as well as gaps in SGRP data have *phred *quality scores [8] (according to this standard, each quality character is translated into the probability of error for that read. For instance, a character "A" means that the corresponding read is wrong with the probability of error p = 10^-3.2^ = 0.00063). We filter out all data reads that have a quality score less than 40 (i.e. those with a probability of error more than 10-^4^) as well variations that occur in yeast genes annotated as dubious in SGRP. To identify indels with a better quality, we smoothed out the quality scores of the indels with their neighbouring reads (2 each side). We then marked indels with frequency ( < 2) as missing data. Then a simple heuristics algorithm was applied to remove gaps that are due to misalignments of repetitive short elements in genes. It is commonly observed that a FS indel is followed by a stop codon (created due to the frame-shift). We excluded these stop codons from our analysis of NMs and confirm that all our NMs are due to a single nucleotide NMs. In selecting mutant genes, we require that at any event of indel or NM, there is at least 10 high-quality reads available.

In our *S.cer *dataset, we identified 71 genes with single FS indels and 96 genes with single NM throughout the genome data for which the ancestral state were known. Due to sparse PWMs, we excluded 14 (20%) genes with FS indels and 22 (23%) genes with NMs from the analysis. We then predicted the effect of 57 genes with single frame-shifting indel and 74 genes with a single NM. Analysis of genes with more than one form of variation requires considering mutual effects of variations on function that is the subject of our near future research.

### Measuring conservation in yeast non-coding DNA

We aligned protein-coding sequences of 5140 genes in *S.cer *with their orthologues from 12 other yeast species. The evolutionary tree of these species is as in the following:

For each gene, the corresponding alignment was used to estimate the evolutionary tree, T, using Felsenstein peeling algorithm [48]. The estimated tree was then used to compute the synonymous rate of evolution λ_syn _using CodeML (part of PAML suite of software [57]) for that gene. In computing the denominator of the LLR, we used evolutionary tree, T, as well as the synonymous rates, λ_syn_, to compute the likelihood of each DNA site.

We identified 6198 indels with maximum length of 50 bps in the 1000 bp DNA upstream of 5140 genes in our dataset. We were able to make alignments for 4710 of these variants and excluded 1488 (24%) of the indels from analysis due to sparse alignments, and excluded further 462 which had frequency < 2. For each indel, we consider a window of width W = 21 centred at it. We refer to this short stretch of bases as the DNA site. For each such site, we made an alignment of orthologue sequences (see Figure 2) of 5 closely related yeast species:

We then used these alignments to estimate the rate of evolution of the DNA site, , using BaseML in PAML [57]. In computing the maximum likelihood estimate of the rate, we fixed the structure of the synonymous tree, T, and maximized the likelihood of the alignment by incrementally changing the size of the tree branch lengths while keeping their ratio constant.

The result of the algorithm is summarized as a list of indels with their corresponding DNA sites (width W = 21) ranked by their conservation (LLR score). The sites with highest LLR scores consist of fully conserved DNA bases in all W columns of 5 sequences (Figure 2).

### Finding a threshold for deleterious mutations using Poisson random field

We observed that indels that fall in highly conserved DNA sites tend to segregate in lower derived allele frequencies. To test this observation quantitatively, we defined a threshold THR and classified the mutations into two classes by comparing their LLR score with the threshold. Mutations in the class with (LLR > THR) were expected to segregate in comparably lower derived allele frequencies (i.e. the spectrum of their mutations were expected to shift more towards lower allele frequencies [8,23]).

To measure the difference between the spectrums of allele frequencies for these two classes, we fit two separate Poisson random fields [58] on each class of mutations and computed corresponding selection coefficients for each class using maximum likelihood estimation [59].

Each value of the threshold, therefore, results in two classes of mutations with two different selection coefficients. We then repeated the experiment for different values of the threshold to identify an optimal threshold that results in maximal difference between selection coefficients for the two classes.

By dividing the mutations based on their LLR scores and using the optimal threshold we obtained two classes of mutations that have maximal difference in their derived allele frequency spectrum. The maximum and minimum LLR scores in our dataset were 40.6 and 0, respectively. We estimated an optimal threshold of THR = 16.6. We used this threshold to obtain derived allele frequency spectrums as shown in (Figure 3e).

A Similar approach was used to determine the threshold for the information loss score D associated with NMs in the human population. The mutations used in this analysis had no record of disease association and therefore included mutations with lower deleterious effects (there was no genotype information for NMs with records of disease association). We divided these mutations into two classes by comparing their score D with a threshold THR. We then obtained the THR similar to what is explained in the above. We obtained THR = 0.3 for the NMs in the human population (Figure 3f)

### Variations in the human population

We used all NMs and FS indels reported in the release 132 of dbSNP [10] for our human variations dataset. We used NCBI API to fetch the data from dbSNP. There are 33285 (8311) FS indels (NMs) reported in dbSNP from which 801 (2234) FS indels (NMs) have records of diseases association in OMIM [11] and LSDB [12]. No filtering was done to ensure that the variations were unique, and we noted that ~25% of NMs and FS indels seem to be listed under multiple mutation identifiers.

We were able to compute the information loss score D for 2047 of the 2234 (91%) NMs with disease association records. We randomly chose 4468 (2234 × 2) NMs from the set of 8311 NMs with no records of disease association from which we were able to compute the D score for 3816 (85%) NMs. There was no heterozygocity information available for NMs with disease association. Therefore were able to compute the minor-allele frequency (MAF) of 1606 NMs in the dataset with no record of disease association.

For the case of FS indels, we computed the information loss score D for 696 (86%) of the 801 indels with disease association records. We randomly selected 1602 (801 × 2) indels from the set of 6317 indels with no disease association from which we were able to compute the D score for 1312 (81%) indels. There was no heterozygocity information available for the FS indels.

On average, we excluded 14% of human variants from our analysis due to sparse PWMs.

For computing the minor-allele frequency (MAF) of mutations (q), we used the heterozygocity (h) and solved the equation h = 2q(1-q). Our MAF spectrum analysis is based on the data for the NMs with no disease association record in OMIM and LSDB.

## Competing interests

The authors declare that they have no competing interests.

## Authors' contributions

AZ performed the analysis and analyzed the data, AZ designed the method, AMM and AZ wrote the manuscript and AMM conceived of the project and supervised the research. All authors read and approved the final manuscript.

## Supplementary Material

Additional file 1 **Supplementary_Figures.pdf** Supplementary figures S-1 and S-2 in PDF format. Click here for file
