# Supplementary material for: Ranking insertion, deletion and nonsense mutations based on their effect on genetic information
Source: BMC Bioinformatics. 2011 Jul 22;12:299. doi: 10.1186/1471-2105-12-299 (PMC3155974; doi:10.1186/1471-2105-12-299)
Supplement: Additional file 1 — Supplementary_Figures.pdf Supplementary figures S-1 and S-2 in PDF format. [file 1471-2105-12-299-S1.PDF]

Supplementary figures

Ranking insertion, deletions and non-sense mutations based on their effect on genetic information

Amin Zia, Alan M. Moses

A nonsense mutation (NM) truncates a protein and a frame-shifting (FS) indel causes a mistranslation of the amino acids from the position of the indel forward. In the following, we refer to fraction of the protein that is truncated in the NM and the portion that is mistranslated in the FS as the "length loss" score.

Figure S1 – Scatter plot of genes with mutations in terms of the information loss score D versus corresponding length loss.

(a-d) The information loss score caused by mutations is plotted against corresponding length loss for the FS-indels and the NMs in the yeast and the FS-indels and the NMs in the human dataset, respectively. The score D is strongly correlated with the length loss caused by mutations (R=0.945 on average). In these figures there are several cases of off the diagonal where the length lost does not correlate with the D score and therefore reflects the effect of the mutation differently. Although these are a minority of cases, we believe that in these cases the D score represents a more principled approach that takes into account the underlying evolutionary effects on proteins.

Figure S2 - Randomization experiments (position of mutations)

Distributions of genes with respect to the length loss are compared with random expectations. For the randomization tests, we randomly distributed mutations over the proteins in our datasets uniformly and recorded the length lost (shown in this figure) and measured the loss of information score D (shown in Figure 4). We repeated the randomizations for 100 for yeast indels and NMs and 50 times for other comparions. The error bars show one standard deviation from mean (two standard deviations in total). This figure is in comparison with Figure 4. (a) Distribution of yeast genes with respect to the length loss caused by the FS indels in the yeast population (blue) compared to the density of a set of randomly distributed FS indels throughout the same set of yeast genes (red). (b) Distribution of yeast genes with respect to the information loss caused by the NMs in the yeast population (blue) compared to a set of randomly distributed NMs throughout the same set of yeast genes (red). (c-d) Distributions of FS indels and NMs in the human population, respectively, with respect to the length loss they cause.

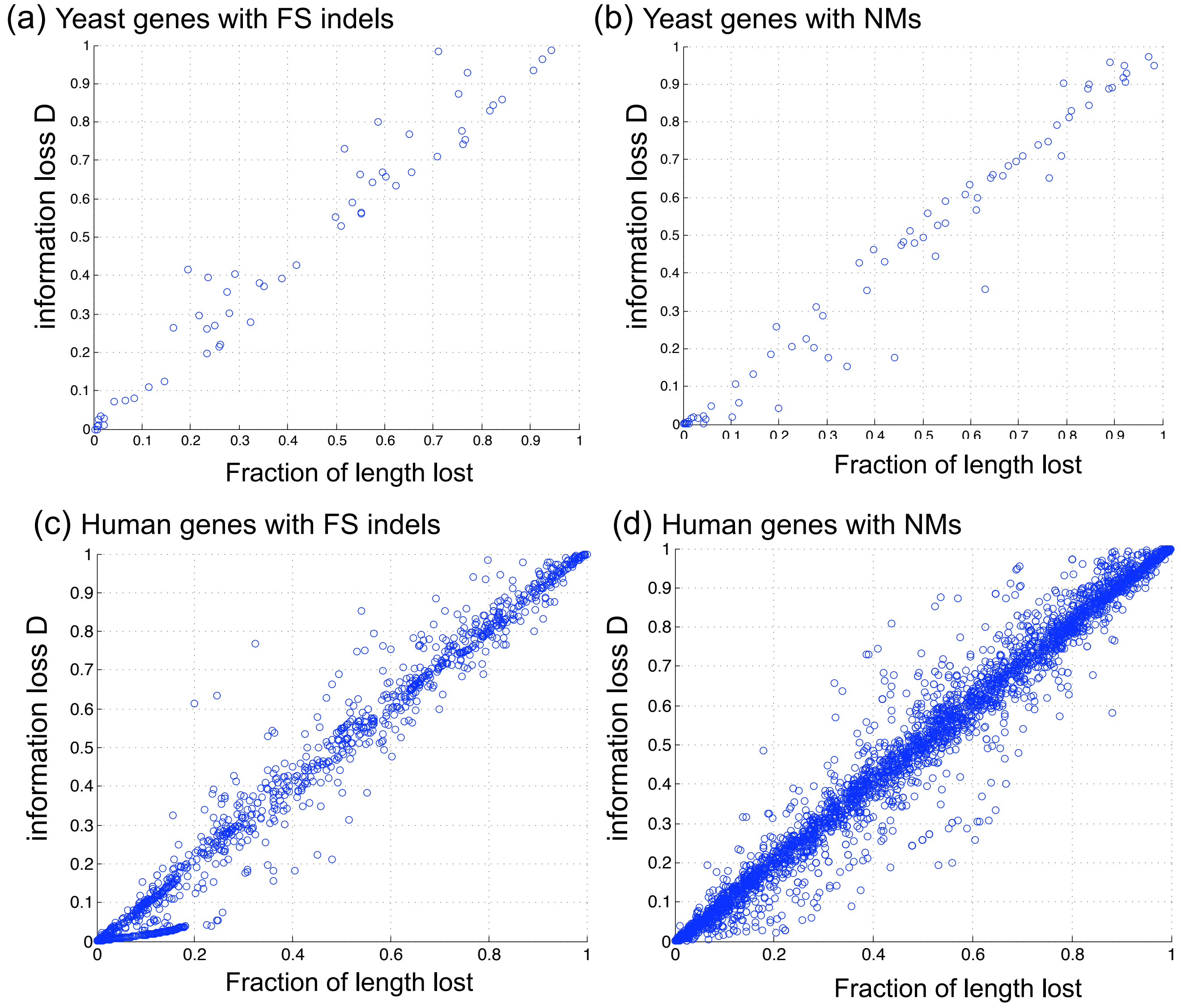
(a) Yeast genes with FS indels
(b) Yeast genes with NMs
(c) Human genes with FS indels
(d) Human genes with NMs
information loss D
Fraction of length lost

Figure S1

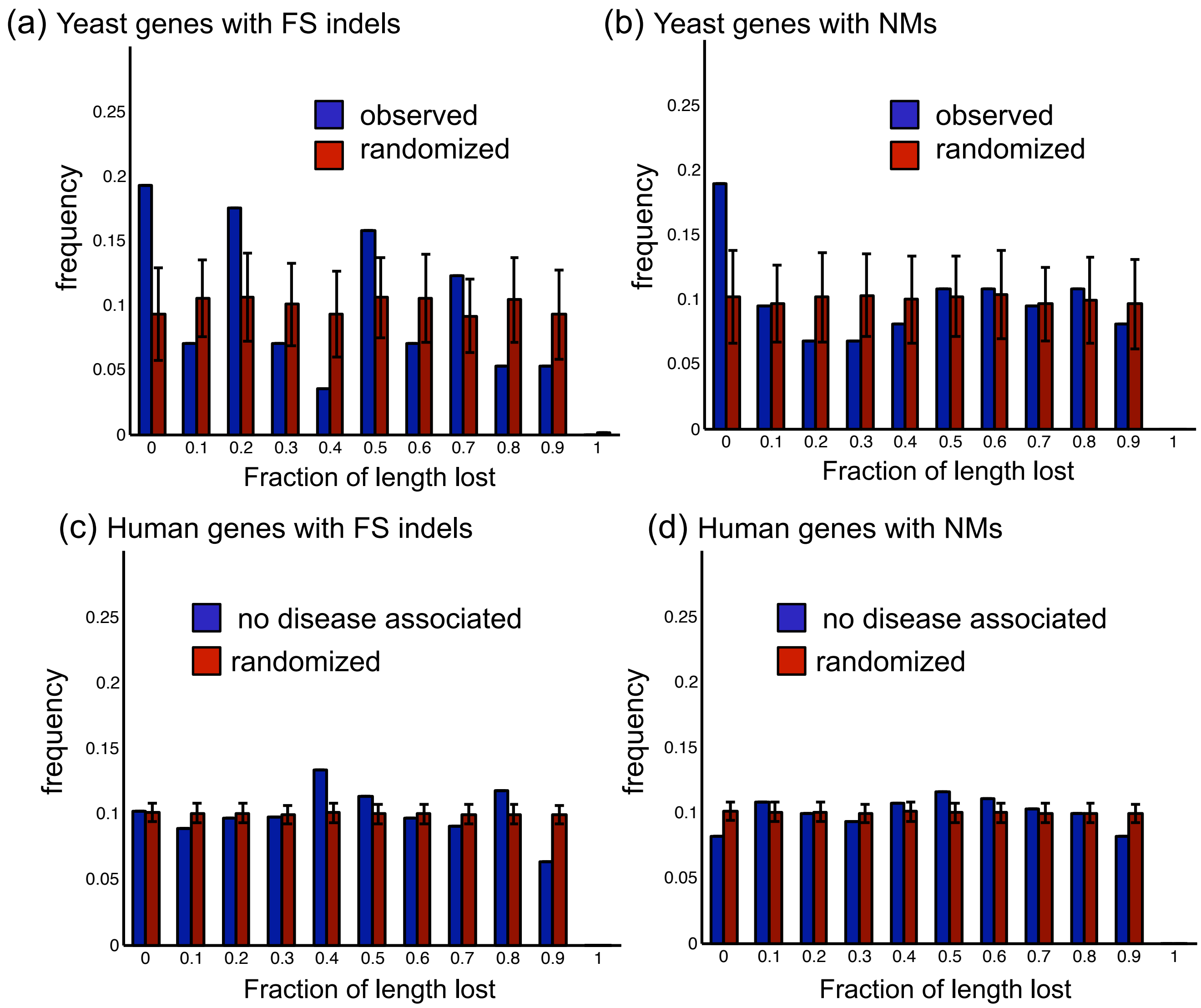
(a) Yeast genes with FS indels
observed
randomized
frequency
0.25
0.2
0.15
0.1
0.05
0
0 0.1 0.2 0.3 0.4 0.5 0.6 0.7 0.8 0.9 1
Fraction of length lost
(b) Yeast genes with NMs
observed
randomized
frequency
Fraction of length lost
(c) Human genes with FS indels
no disease associated
randomized
frequency
Fraction of length lost
(d) Human genes with NMs
no disease associated
randomized
frequency
Fraction of length lost

Figure S2
